# Association between Urinary Aflatoxin (AFM_1_) and Dietary Intake among Adults in Hulu Langat District, Selangor, Malaysia

**DOI:** 10.3390/nu10040460

**Published:** 2018-04-07

**Authors:** Siti Husna Sulaiman, Rosita Jamaluddin, Mohd Redzwan Sabran

**Affiliations:** Department of Nutrition and Dietetics, Faculty of Medical and Health Sciences, Universiti Putra Malaysia, 43400 Serdang, Selangor, Malaysia; sitihusna11@gmail.com (S.H.S.); mohdredzwan@upm.edu.my (M.R.S.)

**Keywords:** aflatoxin, aflatoxin M_1_, urinary AFM_1_, dietary intake, Hulu Langat, Malaysia

## Abstract

Aflatoxin is a food contaminant and its exposure through the diet is frequent and ubiquitous. A long-term dietary aflatoxin exposure has been linked to the development of liver cancer in populations with high prevalence of aflatoxin contamination in foods. Therefore, this study was conducted to identify the association between urinary aflatoxin M_1_ (AFM_1_), a biomarker of aflatoxin exposure, with the dietary intake among adults in Hulu Langat district, Selangor, Malaysia. Certain food products have higher potential for aflatoxin contamination and these were listed in a Food Frequency Questionnaire, which was given to all study participants. This allowed us to record consumption rates for each food product listed. Concomitantly, urine samples were collected, from adults in selected areas in Hulu Langat district, for the measurement of AFM_1_ levels using an ELISA kit. Of the 444 urine samples collected and tested, 199 were positive for AFM_1_, with 37 of them exceeding the limit of detection (LOD) of 0.64 ng/mL. Cereal products showed the highest consumption level among all food groups, with an average intake of 512.54 g per day. Chi-square analysis showed that consumption of eggs (*X*^2^ = 4.77, *p* = 0.03) and dairy products (*X*^2^ = 19.36, *p* < 0.01) had significant associations with urinary AFM_1_ but both food groups were having a phi and Cramer’s V value that less than 0.3, which indicated that the association between these food groups’ consumption and AFM_1_ level in urine was weak.

## 1. Introduction

Foodborne disease is a global concern, as the Centers for Disease Control and Prevention [1] estimates that each year there are 48 million people who are affected, 128,000 of whom are hospitalized and 3000 die. Foodborne diseases are commonly caused by bacteria, viruses, parasites, harmful toxins and chemicals. Mycotoxins, one of the etiologic agents of foodborne diseases, are produced by fungi which can cause mycotoxicosis [2]. There are many mycotoxins that have the potential to contaminate food products and agricultural commodities. Aflatoxin is produced by *Aspergillus* species of fungi such as *Aspergilllus flavus*, *A. parasiticus* and *A. nomius* [3]. These fungi are found in the warm and humid climates prevailing in the tropical and sub-tropical geographic latitudes [4]. In addition, improper food storage and production procedures promote the growth of these fungi, and subsequently aflatoxin contamination, in many agricultural commodities.

The most common aflatoxin metabolites found in foodstuff are aflatoxin B_1_ (AFB_1_), aflatoxin B_2_ (AFB_2_), aflatoxin G_1_ (AFG_1_), and aflatoxin G_2_ (AFG_2_) [5]. Of these, AFB_1_ is the most toxic as it has been classified by the International Agency for Research on Cancer (IARC) as Group 1 carcinogen [6]. Besides their occurrence in the foods and agricultural commodities, aflatoxin can be detected in biological samples, resulting from exposure through the diet. In fact, the assessment of human and animal exposure to aflatoxin, through the detection of aflatoxin biomarkers in biological samples such as in serum and urine, is significant to determine the extent and rate of aflatoxin exposure.

The metabolism of aflatoxins, particularly AFB_1_ in the liver produces several metabolites such as AFB_1_-lysine adduct [7], AFB_1_-N^7^-guanine adduct [8], and urinary AFM_1_ [9], and these biomarkers have been used in many epidemiological studies reported in the literature [7,8,9,10] assessing the extent of human exposure to aflatoxin. For example, the detection of urinary AFM_1_ is a good indicator to determine recent aflatoxin exposure of 1–3 days. In addition, Zhu et al. [10] found a good correlation between total dietary AFB_1_ and the excretion of AFM_1_ in urine.

In Malaysia, the occurrence of aflatoxin in foodstuffs such as cereals, peanuts, spices and their products has been reported. In a review by Mohd-Redzwan et al. [11], on the occurrence of aflatoxin in Malaysia, the authors highlighted the use of aflatoxin biomarkers as a potential tool to provide a better assessment of aflatoxin exposure as different individuals might be exposed at different rates. Indeed, it is of great significance to find the association between aflatoxin biomarkers and human dietary consumption to reflect aflatoxin exposure from the diet. Hence, this research was conducted to determine the association between a urinary AFM_1_ biomarker and food consumption among adults in Hulu Langat district, Selangor, Malaysia.

## 2. Materials and Methods

### 2.1. Study Respondents

The study respondents, including both male and female, ranged in age from 18 to 60 years old, and resided in Hulu Langat district, which is the fifth largest (out of nine total districts) in Selangor, Malaysia. This district consists of six sub-districts (Beranang, Cheras, Hulu Langat, Hulu Semenyih, Semenyih and Kajang). Hulu Langat district was chosen due to desirable density of ethnic population and its proximity to Universiti Putra Malaysia (UPM). A total of 468 respondents underwent a screening examination that included a medical history. All of them gave informed consent and the study was approved by the Ethics Committee for Research Involving Humans at UPM (FPSK (EXP16) P047).

Of the 468 screened respondents, 455 met the following study criteria: (1) in good health; (2) not taking any medications or supplements; (3) not smoking; (4) not following a restricted diet; and (5) not pregnant and not in postpartum period. For the analyses conducted in the current study, 11 respondents were excluded for the following reasons: missing FFQs (*n* = 5), aged more or less than the required range (*n* = 2), and non-returnable urine container (*n* = 4).

### 2.2. Dietary Assessment

Dietary consumption data were collected using an FFQ [12] and took five months to compile. The elaboration of food groups in the FFQ was obtained from previous studies [4,13]. All respondents completed a self-administered FFQ that consisted of 197 food items that are susceptible to aflatoxin contamination (e.g., cereals products, nuts and legumes, eggs, and dairy products) [4,13]. Typical portion sizes for each food item are considered medium-sized, based on the Malaysian Food Serving Size Album [14] and the list of food item weights in household measures [15]. The amount of food intake (g/day) was calculated based on Norimah and colleagues’ formula [12].

For further statistical analysis such as chi-square analysis, all food groups were divided into two groups, low and high, based on the median total of intakes.

### 2.3. Urine Analyses

Fifteen-milliliter morning urine samples were collected in containers, and then delivered to UPM on the same day using a specific ice box. These samples were kept frozen at −80 °C in the Nutrition Laboratory 3, Faculty of Medicine and Health Sciences, UPM until their analyses began.

Quantification of urinary AFM_1_ was undertaken following the protocol of Mohd-Redzwan et al. [16]. The level of AFM_1_ was analyzed using an ELISA kit specifically designed for the determination of urinary AFM_1_ (Helica Biosystems, Inc., Santa Ana, CA, USA). The debris and precipitate were removed through centrifugation at 3000× *g* for five minutes (Kubota Centrifuge Model 2810, Tokyo, Japan), and supernatant was used for the determination of AFM_1_ according to manufacturer instructions. The protocol’s washing step involved the use of an automated microplate washer (Drop ELISA Washer, RADM, Roma, Italy). A microplate reader (SIRIO S Microplate Reader, RADM, Roma, Italy) was used to measure absorbance at a wavelength of 450 nm. As for method validation, urine samples were spiked with 1.5 ng/mL AFM_1_ standard and the samples were processed as per protocol.

Before statistical analyses were conducted, the limit of detection (LOD) was calculated based on the lowest concentration of AFM_1_ obtained from the measurement of urine samples. The concentrations were first extrapolated from the standard curve of AFM_1_. The *r^2^* of the standard curve was 0.998 and LOD was calculated using the formula by Shrivastava and Gupta [17]:

LOD = 3.3*σ*/*s*

Where, *σ* is the standard deviation of the y-intercept of the regression line and *s* is the slope of calibration curve.

The calculated LOD was found to be 0.64 ng/mL. The AFM_1_ readings were then segregated into two groups, positive and negative, based on the presence or absence of detectable AFM_1_. Then, Chi-square statistical analysis was conducted for each reading. Negative samples were categorized as simple with no detectable level of AFM_1_, whereas positive samples were categorized as samples with detectable level of AFM_1_.

### 2.4. Statistical Analysis

Basic demography characteristics were presented as mean ± standard deviation when the data were continuous, and they were presented in contingency tables if the data were binary or categorical. The intake of potentially aflatoxin-contaminated food was presented as average grams per day (g/day). Our use of Chi-square was to determine the association between urinary AFM_1_ biomarker level and dietary intake. The level of significance was accepted as *p* < 0.05. The strength of association between variables was determined through Phi and Cramer’s V value [18].

## 3. Results

### 3.1. Socio-Demographic Characteristics

Table 1 represents the socio-demographic characteristics of respondents. Out of 444 respondents, 246 were females and 198 were males. Most of the respondents were single (*n* = 323, 72.1%) with a median age of 24 years old. About half of the respondents were Chinese (*n* = 211, 47.5%) and most of them had tertiary educational level. Besides, most of the respondents had personal income of less than RM1500 (USD 384.87) while the total household income was RM3500 (USD 898.03) and above.

### 3.2. Dietary Intake

Table 2 represents the median intake based on food group. Cereal products were consumed at the highest rate, while eggs had the lowest intake rate, among the respondents.

Table A1 (Appendix A) shows the means, standard deviations, medians as well as ranges for the daily intakes of potentially aflatoxin-contaminated foods assessed by the FFQs. The top three highly consumed food items were white rice (270.79 g/day), flavored rice (40.54 g/day) and chocolate-flavored milk (38.11 g/day). The two rice items come from the cereal products food group, and the milk is an item from the dairy product food group.

### 3.3. Urinary AFM_1_ Biomarker Level

Negative samples were categorized as samples with no detectable level of AFM_1_, whereas positive samples were categorized as samples with detectable level of AFM_1_ through the extrapolation from the standard curve. Of 444 analyzed urine samples, 199 samples were positive for AFM_1_ while the rest (*n* = 245) were negative. From those with positive AFM_1_, 37 of them exceeded the limit of detection (LOD) of 0.64 ng/mL. Those 37 samples had urinary AFM_1_ ranging from 0.65 to 5.34 ng/mL, with an average of 1.23 ng/mL.

### 3.4. Association between Urinary AFM_1_ Biomarker Level and Socio-Demographic Factors and Dietary Intake

Our findings (Appendix Table A2) indicate several significant association between socio-demographic factors, ethnicity (*p* < 0.01), age (*p* < 0.05) and household income (*p* < 0.01), and urinary AFM_1_ among respondents in Hulu Langat district, Selangor, Malaysia. There were also weak associations between urinary AFM_1_ and consumption of eggs (*X*^2^ = 4.77, *p* = 0.03) and dairy products (*X*^2^ = 19.36, *p* < 0.01), whereas no other food groups showed any significant associations (Table 3).

## 4. Discussion

Aflatoxins are present ubiquitously in nuts and nuts products, cereals, and spices and herbs, which are widely used among Malaysians as the main ingredients in cooking [3]. Milk and dairy products as well as eggs and meat products are contaminated as animals consume aflatoxin-contaminated feed. The rate of aflatoxin contamination in foodstuffs varies from one products to another. A study in Terengganu, Malaysia [22], found that 19 out of 53 dairy products samples were positive with AFM_1_, ranging from 3.5 to 100.5 ng/L, and this contamination levels were still safe according to Malaysian Food Regulation 1985 (>50 ng/L) [23]. Malaysians are one of the Asian citizens that consume rice frequently, as a staple food [24]. Soleimany et al. [19] found detectable levels of aflatoxin, ranging from 0.19 to 3.96 ng/g, in rice sold in the Malaysian General Market in Kuala Lumpur. Besides, there was also a study [20] that found that a maize and two rice samples from the Malaysian markets had aflatoxin levels exceeding the European regulatory limits for aflatoxin, i.e., 4 ng/g. Other than that, another study by Shahzad et al. [25] discovered about 35% of the rice samples was positive with aflatoxin contamination, where brown rice showed the highest aflatoxin contamination (12.4 µg/kg). Another foodstuff that is easily contaminated by aflatoxin is nuts and legumes. A study conducted in Penang, Malaysia [21] found 32 out of 196 nuts and its based products samples were positive with aflatoxin ranging from 16 µg/kg to 711 µg/kg for total aflatoxin and this range exceeded the limit value set of 5 µg/kg for all foodstuffs and 15 µg/kg for processed groundnuts based on the Malaysian Food Regulation 1985. On the other hand, the contamination of aflatoxin in egg samples around Malaysia was less reported but its occurrences do exist in other countries. For example, there was a report that documented the presence of aflatoxin in 22 out of 80 eggs samples collected from central areas of Punjab, Pakistan [26]. The level of aflatoxin B_1_ reported ranged from 0.5 to 3.19 µg/kg for farm eggs and 0.5 to 1.98 µg/kg for domestic eggs. In fact, the level of aflatoxin in eggs commodity depends on the level of other microorganisms on the eggs such as *Salmonella* spp., *Candida* spp. and coccidiosis organisms [27]. As the level of these microorganisms increases, the level of aflatoxin in eggs decreases.

The contamination of food commodities by aflatoxin B_1_, the most toxic of aflatoxin is common in countries with subtropical and tropical climate. Aflatoxin M_1_ (AFM_1_) is a metabolite of AFB_1_ which can be detected in the urine and milk of exposed animals and humans. According to Zhu et al. [10], 1.23–2.18% of ingested AFB_1_ is found as AFM_1_ in the urine. The authors found correlation coefficient (*r*) of 0.65 between AFB_1_ intake and AFM_1_ in the urine among population in China. In fact, a preliminary study conducted in Malaysia showed a linear relationship between dietary AFB_1_ exposure and urinary AFM_1_, and the population was exposed to 0.0262 µg/day/kg AFB_1_ through the diet [16]. Other than that, an intervention study [28] also showed a significant correlation between the concentration of urinary AFM_1_ with the consumption of nut-based products (*r* = 0.258) in a probiotic intervention study.

The consumption of aflatoxin-contaminated food was higher in countries from Southeast Asia compared to developed countries from the Western Europe [29] and it shows that the occurrence of aflatoxin is higher in developing countries, where the prevalence of aflatoxin contamination in foods and agricultural product is high [30]. A study conducted in Bangladesh showed a significant difference of AFM_1_ level in two different areas, rural and urban [31]. The authors found that the mean urinary AFM_1_ level was higher among residents in rural areas (99 ± 71 pg/mL) compared to urban areas (54 ± 15 pg/mL). Besides, the highest AFM_1_ found in the study was among residents in the 50–60 years age group. In contrast, the present study found that the highest AFM_1_ levels were among the younger age group residents (≤24 years of age). In Malaysia, a study found that AFB_1_ exposure ranged from 24.3 to 34 ng/kg body weight/day [32]. Another study, by Mohd-Redzwan et al. [33], found that urinary AFM_1_ level in a population in Serdang, Selangor was 18.8 ± 28.6 pg/mL, ranging from 2.4 to 100.4 pg/mL, which also differs from our findings (average = 1.23 ng/mL). Urinary AFM_1_ level among Chinese respondents were 3.20 times higher compared to non-Chinese respondents [21]. Their findings were comparable to a previous study in Penang conducted by Leong et al. [21], which reported high occurrence of aflatoxin among Chinese respondents was related to the higher intake of food commodities that have high risk of being contaminated by aflatoxin-producing fungi such as cereal, eggs, nuts and legumes and dairy products. Leong et al. [21] further added that food preferences based on cultural differences and food choices may be the cause of the significantly high level of aflatoxin biomarkers.

In our study, 199 respondents had positive occurrence of urinary AFM_1_, who primarily consumed cereal-based products such as white rice (270.77 ± 197.96 g/day). This finding in agreement with the average intake of white rice among Malaysians of 275.03 g/day as reported by the Food Consumption Statistics of Malaysia [34]. Hence, it is postulated that high consumption of cereals, especially white rice, could be associated with high percentage of AFM_1_ occurrence in urine among the respondents. For example, previous studies reported a significant correlation between the occurrence of aflatoxin biomarkers in humans and consumption of cereal-based products [35,36]. In the present study, no significant association was detected between urinary AFM_1_ with the consumption of cereal products. However, respondents who consumed more than 22.71 g/day of egg were more likely to have positive occurrence of AFM_1_ in the urine, as shown in Table 3.

To the best of our knowledge, no associations have been reported in the literature on the intake of eggs and the occurrence of aflatoxin biomarkers in biological samples such as in urine and serum. The presence of aflatoxin metabolite in eggs can be explained when the poultry animals and birds are feeding on aflatoxin-contaminated feed. Aflatoxins are then metabolized by the liver, where the end-products of the metabolism such as AFB_1_-lysine adduct [7], AFB_1_-N^7^-guanine adduct [8], and urinary AFM_1_ [9] are produced. Aflatoxin contamination of eggs has been reported [37]. In fact, the first aflatoxicosis case related to aflatoxin contamination was reported in turkeys and ducklings in the 1960s, where millions died as a result of consuming AFB_1_-contaminated feeds [38].

The present study also found significant association between dairy product consumption and occurrence of urinary AFM_1_ among respondents. Respondents whose daily intake of dairy products exceeded 77.68 g/day were determined to be more likely to have detectable levels of urinary AFM_1_. This finding in agreement with a study conducted by Mohd-Redzwan et al. where the level of urinary AFM_1_ was significantly higher (2.67 ± 2.27 ng/mL) among respondents with high intake of dairy products than those with low intake [16]. In Malaysia, the permissible level of aflatoxin in food and agricultural commodities is regulated in Malaysian Food Regulations 1985 [23] which permits a maximum level of aflatoxin in dairy products of 0.5 µg/kg, similar to the maximum level suggested by Codex Alimentarius Commission (CAC) for the international reference [39].

Although the other food groups examined in this study did not show significant association with the occurrence of urinary AFM1, it should be noted that these food groups (e.g., cereal-based products, and nuts and legumes) are susceptible to fungal infection and aflatoxin contamination. Most of the occurrences were related to the consumption of aflatoxin-contaminated staple food. For example, the largest outbreak of human aflatoxicosis reported so far occurred in Kenya during 2004 [40]. This case reported as high as 8000 µg/kg of aflatoxin in maize and this contamination led to 125 deaths. Other than maize, rice is another staple food cultivated in subtropical areas that is highly exposed to aflatoxin. The Food and Agriculture Organization (FAO) reported that 15% of the harvested rice is damaged annually due to poor storage conditions, which encourage the growth of mycotoxin-producing fungi [41]. Ephrem [42] found that contamination of aflatoxin in rice was as high as 2830 µg/kg, which is higher than the levels of contamination in wheat and maize, as reported in several cases. Ephrem [42] also emphasized that some of the foods may lack of pre-harvest storage practice. Thus, the growth of aflatoxin-producing fungi occurs and allows aflatoxin contamination. In addition, food groups such as nuts and legumes are suitable for the growth of aflatoxin producing fungi due to high fat contents of this food group [42]. A study in Malaysia found that 16.33% of the collected nuts and nut-based products samples were contaminated by aflatoxins, ranging from 16.6 µg/kg to as high as 711 µg/kg [43]. This level of contamination is higher than several studies conducted in Pakistan (less than 15 µg/kg) [44] and Iran (less than 16 µg/kg) [45].

Most mycotoxins are stable during food processing [46] so it can even show up in food products such as peanut butter and many processed products. However, certain food processing techniques can reduce the toxicity of aflatoxin in food products through physical removal and chemical or enzymatic decontamination [46]. That must be one of the reasons for the significant association between AFM_1_ levels and the consumption of eggs and dairy products discovered from this study. The highest consumption of food from food group of eggs and dairy products were hen eggs (mean ± standard deviation = 33.51 ± 44.64) and chocolate-flavored milk (mean ± standard deviation = 38.07 ± 92.71). Both food items are subjected to one or more processing steps before consumption. For example, the processes involved in producing flavored milk are pasteurization, sterilization and boiling, which reportedly can reduce the concentration of aflatoxin in milk [47]. Previous studies also mentioned that boiling, sterilization and pasteurization reduce aflatoxin in dairy products by 14.5%, 12.2% and 7.6%, respectively [48,49]. Eggs also undergo pasteurization and cooking processes such as boiling, frying, poaching, and steaming before they are consumed. Thus, the aflatoxin concentration in eggs can be reduced too. Food processing that applies heat such as boiling, roasting and steaming can reduce aflatoxin in food by 50–70% [50]. These high temperature processes are not the best solution to reduce aflatoxin levels in certain foods, such as legumes as high heat can deteriorate their nutrients and vitamins.

## 5. Conclusions

Results from this study indicate a low exposure of AFB_1_ in Hulu Langat district, Selangor, Malaysia through the detection of urinary AFM_1_. Future study is encouraged to focus on broader areas which cover the whole of Malaysia as well as to examine the extent of aflatoxin exposure among the general population of Malaysia. The measurement of the AFM_1_ in urine samples indicated recent exposure of aflatoxin AFM_1_ within the preceding 24 h. Since this biomarker is short-lived, and its measurement in urine samples may vary from day to day as well as the dietary intake of the individual, it may not be suitable for assessing long-term aflatoxin exposure. Thus, it is suggested to use other biological samples such as serum AFB_1_-lysine adduct and hair, which can represent the long-term effect of aflatoxin exposure [51].

## Figures and Tables

**Table 1:** Socio-demographic characteristics of respondents (*n* = 444).

Socio-Demographic		*n*	%
Gender	Male	198	44.6
	Female	246	55.4
Marital status	Single	320	72.1
	Married	116	26.1
	Divorced	4	0.9
	Others	4	0.9
Ethnic	Malay	84	18.9
	Chinese	211	47.5
	Indian	147	33.1
	Others	2	0.5
Educational level	No education	3	0.7
	Primary	9	2.0
	Secondary	107	24.1
	Tertiary	325	73.2
Personal income	≤RM1500	300	67.6
	RM1501–RM3499	115	25.9
	≥RM3500	29	6.5
Household income	≤RM1500	130	29.3
	RM1501–RM3499	156	35.1
	≥RM3500	158	35.6
Age ^1^	29.25 ± 11.87		

^1^ Presented as mean ± standard deviation.

**Table 2:** Median of food intake for each group.

Food Groups	Median
Cereal products	452.68
Eggs	22.71
Nuts and legumes	54.19
Dairy products	77.68

**Table 3:** Aflatoxin occurrences in Malaysians’ foodstuff and the Chi-Square analyses on the association between dietary intake level and AFM_1_ biomarker presence among respondents ^a^.

Group	Aflatoxin Occurrence in Malaysian’s Foodstuff	Median of Total Intake (g/day)	AFM_1_		Pearson Chi-Square (*X*^2^)	Phi-Value Association (*φ*)	*p*-Value
			Positive, *n* (%)	Negative, *n* (%)			
**Cereal products**	-Aflatoxin ranged from 0.19 to 3.96 ng/g in rice sold in the Malaysian General Market in Kuala Lumpur [19] -AFM_1_ levels in a maize and two rice samples from the selected Malaysian markets exceeded the European regulatory limits [20]	≤452.68 (low)	91 (41.2)	130 (58.8)	2.36	−0.07	0.12
		>452.68 (high)	108 (48.4)	115 (51.6)			
**Eggs**	-No recent cases were reported in Malaysia	≤22.71 (low)	93 (39.9)	140 (60.1)	4.77	−0.10	0.03 *
		>22.71 (high)	106 (50.2)	105 (49.8)			
**Nuts and legumes**	-Aflatoxin existed in 32 from 196 nuts and its products samples in Penang, ranging from 16 µg/kg to 711 µg/kg [21]	≤54.19 (low)	99 (44.4)	124 (55.6)	0.03	−0.01	0.86
		>54.19 (high)	100 (45.2)	121 (54.8)			
**Dairy products**	-19 out of 53 dairy products positive AFM_1_, ranging from 3.5 to 100.5 ng/L [22]	≤77.68 (low)	76 (34.4)	145 (65.6)	19.36	0.21	0.00 **
		>77.68 (high)	123 (55.2)	100 (44.8)			

* *p*-value < 0.05, ** *p*-value < 0.01, ^a^ Computed for 2 × 2 table.

## Appendix A

**Table A1:** Intake of aflatoxin-potentially-contaminated food (*n* = 444).

Food Group	Food Items	Mean ± Standard Deviation	Median	Range
Cereal products	White rice ^1^	270.77 ± 197.96	255.09	0–1431.00
	Brown rice	14.27 ± 72.63	0.14	0–1170.00
	Flavored rice (“Nasi briyani”, fried rice) ^2^	40.55 ± 103.32	0.83	0–1150.00
	Rice porridge	5.92 ± 16.51	0.25	0–166.00
	Glutinous rice	3.24 ± 14.46	0.16	0–190.00
	Noodles ^3^	29.72 ± 71.24	15.57	0–981.00
	Bihun/kway teow/laksa/laksam/loh shi fun ^a^	24.70 ± 51.04	11.36	0–590.00
	Pasta	5.33 ± 15.37	0.23	0–163.43
	Sagu/ambuyat/linut ^4,b^	1.80 ± 0.95	0.03	0–172.00
	Bread	25.16 ± 31.68	13.68	0–294.00
	Whole meal bread	8.93 ± 34.27	0.49	0–600.00
	Bread bun	7.83 ± 21.40	1.00	0–270.00
	Roti canai ^c^	27.96 ± 58.94	11.07	0–665.00
	Capati ^d^	7.77 ± 23.60	0.66	0–200.00
	Tosai ^e^	7.27 ± 16.70	0.77	0–160.00
	Breakfast cereal	4.51 ± 12.78	0.09	0–144.00
	Cereal grains prepared by water	6.87 ± 36.62	0.02	0–520.00
	Corn	3.44 ± 11.06	0.09	0–126.86
	Wheat	7.22 ± 31.09	0.23	0–396.00
	Barley	9.29 ± 46.44	0.31	0–624.00
Eggs	Hen eggs ^1^	33.51 ± 44.64	22.71	0–484.57
	Duck eggs ^3^	0.81 ± 7.40	0.02	0–146.00
	Quail eggs ^4^	0.16 ± 1.00	0.01	0–13.71
	Salted egg ^2^	2.02 ± 11.28	0.04	0–194.00
Nuts and legumes	Legumes (Green bean, kacang kuda, red bean)	5.68 ± 18.06	0.09	0–230.00
	Groundnut ^3^	7.39 ± 123.05	0.02	0–2600.00
	Taufufa ^f^	3.00 ± 7.73	0.24	0–70.86
	Taufu ^g,1^	37.89 ± 86.80	0.78	0–868.57
	Fermented soy beans (Tempe)	5.68 ± 20.75	0.12	0–213.00
	Steamed redbean bun	2.30 ± 7.92	0.07	0–80.00
	Peanut butter biscuit/wafer	1.44 ± 4.77	0.02	0–56.00
	Kuih kacang ^h^	0.60 ± 2.89	0.01	0–38.00
	Peanut sauce	2.50 ± 11.45	0.02	0–172.00
	Rempeyek ^i^	0.64 ± 4.85	0.01	0–90.00
	Almond	1.02 ± 4.02	0.02	0–45.00
	Rojak sauce ^j^	1.37 ± 6.45	0.01	0–75.00
	Cashew nut	0.83 ± 3.63	0.04	0–39.60
	Canned braised peanut	0.49 ± 3.82	0.01	0–51.00
	Brazil nut ^4^	0.16 ±1.28	0.001	0–17.50
	Almond biscuit	2.46 ± 8.70	0.05	0–87.00
	Peanut biscuit	0.91 ± 3.18	0.02	0–38.40
	Pistachio	0.32 ± 1.60	0.004	0–22.86
	Chestnut	0.92 ± 9.51	0.02	0–184.00
	Hazelnut biscuit	0.58 ± 3.84	0.004	0–60.00
	Peanut soup	6.44 ± 25.75	0.16	0–215.00
	Ice cream with nuts	4.07 ± 11.54	0.11	0–138.00
	Walnut	0.90 ± 5.19	0.02	0–84.00
	Cashew nut biscuit	0.23 ± 1.62	0.003	0–30.00
	Almond powder	0.24 ± 1.40	0.004	0–15.01
	Roasted hazelnut	0.26 ± 1.54	0.002	0–21.43
	Peanut slice	0.19 ± 1.71	0.002	0–34.00
	Ais kacang/ABC ^k,2^	20.93 ± 68.69	1.12	0–1150.00
	Cake or bread with nuts	3.40 ± 8.59	0.15	0–72.20
Dairy products	Cow’s fresh milk ^2^	32.85 ± 67.36	0.58	0–450.00
	Goat’s fresh milk	2.43 ± 18.10	0.04	0–300.00
	Chocolate flavoured milk ^1^	38.07 ± 92.71	0.52	0–855.04
	Strawberry flavoured milk	12.59 ± 51.19	0.13	0–500.00
	Coffee flavoured milk ^3^	17.70 ± 62.58	0.14	0–500.00
	Full cream yogurt	6.48 ± 35.95	0.66	0–300.00
	Low fat yogurt	8.35 ± 25.27	0.18	0–300.00
	No fat yogurt	1.89 ± 16.21	0.10	0–300.00
	Full cream yogurt with fruits	3.47 ± 23.26	0.20	0–300.00
	Low fat yogurt with fruits	2.51 ± 17.27	0.35	0–342.86
	No fat yogurt with fruits	2.57 ± 21.82	0.17	0–300.00
	Full cream yogurt drink	0.92 ± 4.91	0.06	0–42.86
	Low fat yogurt drink	2.76 ± 16.12	0.31	0–300.00
	Regular powdered milk	2.12 ± 6.08	0.01	0–42.00
	No fat powdered milk	0.12 ± 1.39	0.01	0–28.00
	Evaporated milk unsweetened	13.04 ± 84.81	0.07	0–1342.29
	Cheddar cheese	0.86 ± 3.46	0.01	0–36.00
	Mozarella	0.24 ± 1.30	0.02	0–15.00
	Swiss cheese	0.13 ± 1.60	0.01	0–30.00
	Parmesan	0.18 ± 1.66	0.01	0–30.00
	Ricotta	0.04 ± 0.25	0.02	0–2.14
	Cottage cheese	0.04 ± 0.25	0.01	0–4.29
	American cheese ^4^	0.02 ± 0.14	0.01	0–2.14
	Full cream pasteurized milk	5.13 ± 29.51	0.28	0–450.00
	Low fat pasteurized milk	1.82 ± 12.85	0.14	0–150.00
	High calcium pasteurized milk	1.91 ± 11.51	0.15	0–150.00
	No fat pasteurized milk	0.61 ± 4.42	0.02	0–42.86
	Full cream sterilized milk	1.27 ± 8.90	0.07	0–150.00
	Low fat sterilized milk	2.16 ± 16.34	0.12	0–150.00
	No fat sterilized milk	0.32 ± 2.25	0.01	0–21.43
	Cultured milk (Yakult, Vitagen, Sustgen)	9.69 ± 38.88	0.71	0–535.71
	3 in 1 (Milo, Nescafe, Nestum)	12.12 ± 41.01	0.32	0–560.00
	Sweetened condensed milk	2.35 ± 9.20	0.10	0–76.00

^a^ Bihun/kway teow/laksa/laksam/loh shi fun = variety type of noodles consumed by Malaysian; ^b^ Sagu/ambuyat/linut = starchy food extracted from a tropical palm stems (especially Metroxylon sagu spp.); ^c^ Roti canai = oiled flatbread; ^d^ Capati = wholemeal, flat pancake-like bread; ^e^ Tosai = Indian pancake; ^f^ Taufufa = Soy bean pudding; ^g^ Taufu = soy bean curd; ^h^ Kuih kacang = mung bean fritters; ^i^ Rempeyek = deep-fried savory Javanese cracker; ^j^ Rojak sauce = Spicy, thick, brown sauce with crushed peanut eaten with Malaysian fruits salad; ^k^ Ais kacang/ABC = Sweet shaved ice with beans. ^1^ The highest consumption of food; ^2^ the second highest consumption of food; ^3^ the third highest consumption of food; ^4^ the lowest consumption of food.

**Table A2:** AFM_1_ level based on socio-demographic factors (*n* = 37).

Socio-Demographic Factors	*n*	AFM_1_ Level (ng/mL) Mean± SEM ^a^	*p*-Value ^b^	Range
Gender			0.81	0.65–5.34 ng/mL
Male	14	1.04 ± 0.10		
Female	23	1.35 ± 0.23		
Marital status			0.19	
Married	15	1.31 ± 0.21		
Single/Divorced/Others	22	1.18 ± 0.21		
Ethnic			0.01 **	
Chinese	18	1.16 ± 0.14		
Non-Chinese	19	1.30 ± 0.26		
Educational level			0.62	
Low	13	0.95 ± 0.12		
High	24	1.39 ± 0.22		
Personal income			0.75	
≤RM1500	15	1.00 ± 0.10		
≥RM1501	22	1.39 ± 0.24		
Household income			0.01 **	
≤RM1500	3	0.84 ± 0.16		
≥RM1501	34	1.27 ± 0.16		
Age			0.05 *	
24 and below	27	1.36 ± 0.20		
25 and above	10	0.90 ± 0.05		

^a^ Standard error mean, ^b^ Obtained from Chi-square analysis, * *p* < 0.05, ** *p* < 0.01.
